# Oiling the wheels of growth: TOR kinase promotes lipid synthesis in plants

**DOI:** 10.1093/plphys/kiaf065

**Published:** 2025-03-04

**Authors:** Moona Rahikainen

**Affiliations:** Assistant Features Editor, Plant Physiology, American Society of Plant Biologists; Organismal and Evolutionary Biology Research Program, Faculty of Biological and Environmental Sciences, University of Helsinki, Helsinki FI-00014, Finland

Target of Rapamycin (TOR) is a conserved eukaryotic serine/threonine protein kinase. In plants TOR controls metabolism and growth in relation to external and internal cues, including nutritional factors and light and sugar signaling. Under favorable growth conditions, TOR activates cell growth and division via phosphorylation of E2Fa transcription factor (Xiong et al. 2013). Conversely, TOR negatively regulates the recycling of cell structures via downregulation of autophagy (Liu and Bassham 2010). In addition, in mammals, TOR signaling has been established to control lipid metabolism under nutritionally and energetically favorable conditions. To this end, mTORC1 increases the activity of the transcription factors sterol regulatory element binding protein 1/2 (SREBP1/2) and early growth response 1 (EGR1) (Porstmann et al. 2008; Chakrabarti et al. 2013).

In *Arabidopsis thaliana*, TOR in encoded by a single gene whose loss is lethal for the plant (John et al. 2011). Thus, estradiol-inducible RNAi line*s* have been used to investigate plant TOR signaling (Xiong and Sheen 2012; Caldana et al. 2013). In contrast to mammals, a previous study using long-term suppression of TOR suggested that in plants TOR negatively regulates the accumulation of triacylglycerol (Caldana et al. 2013). Recently in *Plant Physiology*, Liu et al. (2025) set out to solve this contradiction and investigate the role of TOR signaling in lipid metabolism. The authors used short-term activation or inhibition of TOR signaling and monitored the effects on lipid biosynthesis in 3 plant systems: Arabidopsis seedlings, *Nicotiana benthamiana* leaves, and *Brassica napus* suspension cell culture and demonstrated that TOR is a positive regulator of membrane lipids as well as storage triacylglycerol, similar to mammalian cells (Fig.).

**Figure.**
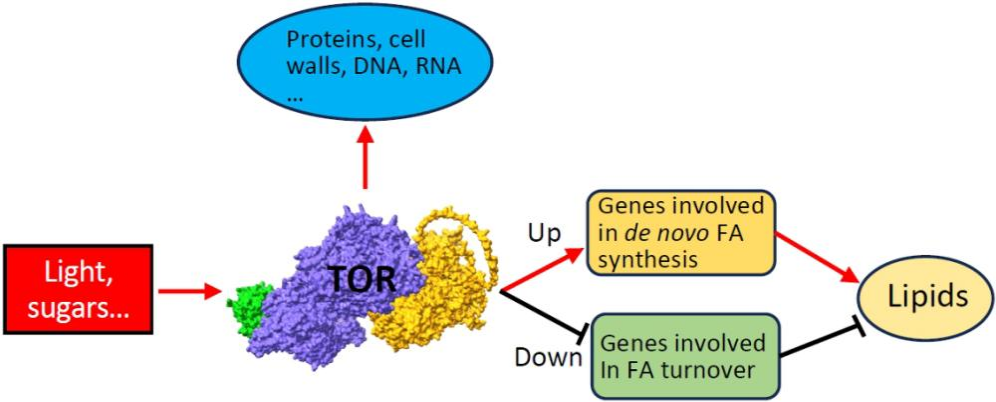
Model of TOR-dependent regulation of plant lipid metabolism. TOR is activated by light and sugar signals and regulates plant lipid metabolism by promoting the expression of genes in de novo fatty acid synthesis and suppressing fatty acid turnover genes. Transcriptional analysis shows that TOR extends its regulation beyond the biosynthesis of proteins, cell wall components, and nucleic acids. The AlphaFold 3 model of TOR complex is depicted and consists of TOR core protein (in purple), LST8 subunit (Lethal with SEC13 protein 8-1, in green) and regulatory protein RAPTOR1B (Regulatory-associated protein of TOR 1B, in yellow). Picture adopted from Liu et al. (2025).

Liu et al. (2025) showed that transient expression of Arabidopsis TOR-GFP under the 35S promoter increased the total fatty acid content in *N. benthamiana* leaves. To support these results, the authors used *tor-es1*, an inducible RNAi line, or Torin 2, a specific ATP competitive inhibitor of TOR (Yuan et al. 2020), to suppress TOR activity in Arabidopsis seedlings. A custom-made antibody was raised to monitor TOR levels during gene silencing. TOR silencing or inhibition by Torin 2 reduced the total fatty acids or fatty acid and membrane lipid content, respectively. In addition, ^14^C-acetate labeling was used to monitor the lipid synthesis rates and confirmed that TOR silencing and Torin 2 inhibited de novo fatty acid biosynthesis. In the tested vegetative tissues, lipids are mostly present in membrane structures, and triacylglycerol content is minute compared with lipid storage organs. To investigate if TOR extends its regulation also to biosynthesis of storage lipids, the authors took advantage of an established model system of *Brassica napus* cv Jet Neuf embryogenic suspension cells that accumulate triacylglycerol up to one-half of their total lipid content (Shi et al. 2008). In line with the findings in Arabidopsis seedlings, Torin 2 also inhibited the accumulation of total fatty acids and triacylglycerol in *B. napus* suspension cells.

In mammals, mTOR1 controls lipid metabolism via transcription factors governing the expression of lipid synthesis enzymes. To investigate the underlying mechanism of TOR-mediated regulation of lipid metabolism in plants, Liu et al. (2025) analyzed Torin 2–induced transcriptional changes after 8 h or 1 day of inhibitor treatment. KEGG pathway and gene ontology enrichment analysis revealed that at the early timepoint protein synthesis and central biosynthetic pathways were upregulated, whereas stress responses were downregulated in mock-treated samples compared with Torin 2 treatment. In the later timepoint, genes associated with biosynthesis of lipids and cell wall components, secondary metabolism and DNA replication were upregulated while stress responses continued to be downregulated. Taking a closer look into lipid metabolism, several genes encoding enzymes in the de novo fatty acid synthesis were downregulated by Torin 2, while several lipolysis genes were upregulated as shown by RNA sequencing and quantitative PCR analyses.

This research clarifies the previous contradictions in the TOR-mediated regulation of plant fatty acid and lipid metabolism and demonstrates that TOR promotes the accumulation of structural lipids and triacylglycerol likely via transcriptional regulation of de novo lipid synthesis and lipid turnover. The authors suggest that previously reported accumulation of triacylglycerol in vegetative plant tissues after prolonged TOR silencing could be a stress response. However, the direct downstream targets of TOR governing lipid metabolism remain to be identified in plants. In sum, this research adds to our understanding of the regulation of plant lipid content and supports the picture of high conservation of TOR signaling in eukaryotes.

## Data Availability

No new data was generated of analysed for this article.
